# Polymorphism of *SERF2*, the gene encoding a heat-resistant obscure (Hero) protein with chaperone activity, is a novel link in ischemic stroke

**DOI:** 10.1016/j.ibneur.2023.05.004

**Published:** 2023-05-10

**Authors:** Andrei E. Belykh, Vladislav O. Soldatov, Tatiana A. Stetskaya, Ksenia A. Kobzeva, Maria O. Soldatova, Alexey V. Polonikov, Alexey V. Deykin, Mikhail I. Churnosov, Maxim B. Freidin, Olga Y. Bushueva

**Affiliations:** a Pathophysiology Department, Kursk State Medical University, Kursk, Russia; b Laboratory of Genome Editing for Veterinary and Biomedicine, Belgorod State National Research University, Belgorod, Russia; c Laboratory of Statistical Genetics and Bioinformatics, Research Institute for Genetic and Molecular Epidemiology, Kursk State Medical University, Kursk, Russia; d Laboratory of Genomic Research, Research Institute for Genetic and Molecular Epidemiology, Kursk State Medical University, Kursk, Russia; e Department of Biology, Medical Genetics and Ecology, Kursk State Medical University, Kursk, Russia; f Department of Medical Biological Disciplines, Belgorod State National Research University, Belgorod, Russia; g Laboratory of Population Genetics, Research Institute of Medical Genetics, Tomsk National Research Medical Center, Russian Academy of Science, Tomsk, Russia; h Queen Mary University of London, London, United Kingdom

**Keywords:** Ischemic stroke, Hero, Heat-resistant obscure, Chaperones, Rs4644832, *SERF2*

## Abstract

**Background:**

Ischemic stroke (IS) is one of the most serious cardiovascular events associated with high risk of death or disability. The growing body of evidence highlights molecular chaperones as especially important players in the pathogenesis of the disease. Since six small proteins called “Hero” have been recently identified as a novel class of chaperones we aimed to evaluate whether SNP rs4644832 in *SERF2* gene encoding the member of Hero-proteins, is associated with the risk of IS.

**Methods:**

A total of 1929 unrelated Russians (861 patients with IS and 1068 healthy individuals) from Central Russia were recruited into the study. Genotyping was done using a probe-based PCR approach. Statistical analysis was carried out in the whole group and stratified by age, gender and smoking status.

**Results:**

Analysis of the link between rs4644832 *SERF2* and IS showed that G allele is the risk factor of IS only in females (OR=1.29, 95%CI 1.02–1.64, Padj=0.035). In addition, the analysis of associations of rs4644832 *SERF2* and IS depending on the smoking status revealed that this genetic variant is associated with an increased risk of IS exclusively in non-smoking individuals (OR=1.26, 95%CI 1.01–1.56, P = 0.041).

**Discussion:**

Sex- and smoking interactions between rs4644832 polymorphism and IS may be related to the impact of tobacco components metabolism and sex hormones on *SERF2* expression.

**Conclusion:**

The present study reveals the novel genetic association between rs4644832 polymorphism and the risk of IS suggesting that SERF2, the part of the protein quality control system, contributes to the pathogenesis of the disease.

## Graphical Abstract

**Figure:**
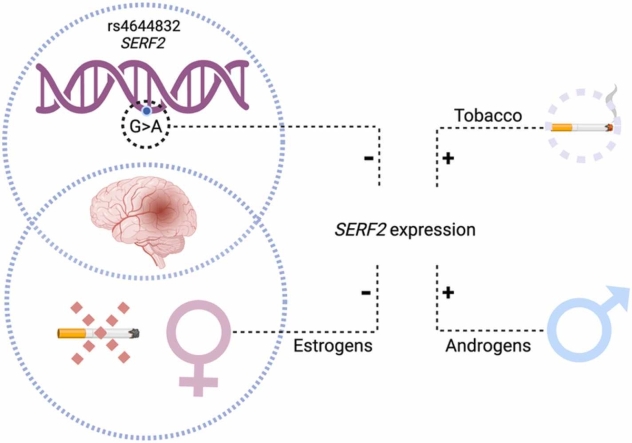

## Introduction

Ischemic stroke (IS) is a life-threatening diagnosis dramatically increasing risk of death and leading to disability in the adult population (Meschia and Brott, 2018). In the vast majority of cases IS is caused by thrombosis following the rapture of the atherosclerotic plaque (Campbell et al., 2019). Study of the mechanisms underlying these events may help to improve approaches for the prediction, prevention and the treatment of the disease ([35], [4], [32], [50], [7]). There are multiple molecular pathways driving the development and outcomes of atherosclerotic plaque in cerebral arteries (Libby, 2021). Foremost, these pathways are linked to cholesterol turnover, vascular wall inflammation and platelets aggregation ([36], [29]). Accordingly, major research has been focused on the certain molecular contributors such as lipoproteins, cytokines and their receptors as well as proteins involved in coagulation and platelets dynamic.

IS is a multifactorial disease with multiple genes involved. Association studies of the last decades revealed a broadest spectrum of genetic markers associated with IS ([1], [25], [55]), and related disorders such as arterial hypertension ([6], [5]) and atherosclerosis ([40], [41], [42], [8]) leading to the new insights in their pathogenesis.

Maintaining proteins is one of the top priority tasks for the cell. Accordingly, chaperones, molecules undergoing the proteins quality control, are the key factors involved in cellular homeostasis. Substantial body of evidence suggests that chaperones serve as important links in genesis and course of IS ([24], [53]). For instance, an imbalance of the 70-kDa heat shock proteins was reported to impede endothelial function (Costa-Beber et al., 2022). Moreover, on preclinical models, modulators of several molecular targets have been tested in various cardiovascular disorders with promising results (Diteepeng et al., 2021).

We set out to investigate whether a recently discovered class of chaperones, so-called “Hero-proteins”, may impact the risk of IS. In brief, Hero is a family of chaperones comprising 6 small heat-resistant proteins. These proteins were shown to protect enzymes from drying, organic solvents and other damaging factors exposed *in vitro* (Tsuboyama et al., 2020). Moreover, some of these proteins also prevented pathological protein aggregation in neural cells during experimental neurodegeneration. Given the critical role of chaperons in atherosclerotic plaque development (Xu et al., 2012) and cellular response to hypoxia (Thiebaut et al., 2019), the Hero proteins may be considered as probable contributors to the risk of IS.

We tested this hypothesis via the analysis of association between the polymorphism of *SERF2* gene encoding a member of the Hero proteins and IS risk in the adult Russian population.

## Material and methods

A total of 1929 unrelated Russians (861 patients with IS and 1068 healthy individuals) from Central Russia were recruited into the study. The study was conducted according to the guidelines of the Declaration of Helsinki, and was approved by the Ethical Review Committee of Kursk State Medical University, Russia (Protocol No. 12 from 7.12.2015). All the participants gave written informed consent before the enrollment in this study, subject to the following inclusion criteria: self-declared Russian descent, a birthplace inside of Central Russia.

Baseline and clinical characteristics of the study population are listed in Table 1. The patients were enrolled into the study in two periods: from the Regional Vascular Center of Kursk Regional Clinical Hospital between 2015 and 2017 and from the Neurology Clinics of Kursk Emergency Medicine Hospital Kursk between 2010 and 2012. All the patients were examined by qualified neurologists. The diagnosis of IS was made in the acute phase of stroke, according to the results of the neurological examination and computed tomography and/or magnetic resonance imaging of the brain. The patients were recruited consecutively. The IS patients were enrolled under the following exclusion criteria: hepatic or renal failure or endocrine, autoimmune, oncological, or other diseases that can cause an acute cerebrovascular event; intracerebral hemorrhage, hemodynamic or dissection-related stroke, traumatic brain injury. All the patients with IS had a history of hypertension and received antihypertensive therapy. Due to the fact that in the studied cohort there were patients with concomitant comorbid pathology, we conducted an additional differential analysis of the baseline and clinical characteristics in subgroups of patients, depending on the presence / absence of ischemic heart disease (IHD) and type 2 diabetes mellitus (T2DM) (Table S1).

**Table 1:** Baseline and clinical characteristics of the studied groups.

Baseline and clinical characteristics		IS patients (n = 861)	Controls (n = 1068)	P
Age, Ме [Q1; Q3]		62 [55; 70]	57 [53; 64]	**< 0.001**
Gender	Males, N(%)	480 (55.7%)	468 (43.8%)	**< 0.001**
	Females, N(%)	381 (44.3%)	600 (56.2%)	
Smoking	Yes, N (%)	429 (49.8%)	318 (29.8%)	**< 0.001**
	No, N (%)	432 (50.2%)	750 (70.2%)	
Coronary artery disease	Yes, N (%)	112 (13.4%)	–	
	No, N (%)	726 (86.6%)	–	
Type 2 diabetes mellitus	Yes, N (%)	95 (11.4%)	–	
	No, N (%)	735 (88.6%)	–	
Body mass index, Ме [Q1; Q3]		23 [22; 26] (n = 561)	ND	
Family history of cerebrovascular diseases	Yes, N (%)	301 (35.92%)	ND	
	No, N (%)	537 (64.08%)	ND	
Age at onset of stroke, Ме [Q1; Q3]		61 [54; 69] (n = 840)	–	
Number of strokes including event in question	1, N (%)	751 (89.5%)	–	
	2, N (%)	76 (9.1%)	–	
	3, N (%)	12 (1.4%)	–	
Stroke localization	Right/left middle cerebral artery basin, N (%)	701 (83.6%)	–	
	Vertebrobasilar basin, N (%)	137 (16.3%)	–	
Area of lesion in stroke, mm^2^, Ме [Q1; Q3]		103 [30; 460] (n = 829)	–	
Total cholesterol, mmol/L, Ме [Q1; Q3]		5.2 [4.4; 5.9] (n = 564)	ND	
Triglycerides, mmol/L, Ме [Q1; Q3]		1.3 [1.1; 1.8] (n = 559)	ND	
Glucose level, mmol/L, Ме [Q1; Q3]		4.8 [4.3; 5.5] (n = 841)	ND	
Prothrombin time, seconds, Ме [Q1; Q3]		10.79 [10.14; 11.7] (n = 826)	ND	
International normalized ratio, Ме [Q1; Q3]		1 [0.93; 1.08] (n = 552)	ND	
Activated partial thromboplastin time, seconds, Ме [Q1; Q3]		32.7 [29.3; 37] (n = 552)	ND	
Alanin aminotransferase, IU/L		21.9 [18; 31.2] (n = 646)	ND	
Aspartate aminotransferase, IU/L		28.2 [20.5; 37.4] (n = 646)	ND	

Statistically significant differences between groups are indicated in bold; ND – no data.

The control group was compiled from healthy volunteers with no clinical evidence of cerebrovascular, cardiovascular, or other chronic diseases and with normal blood pressure without antihypertensive therapy. Healthy individuals were included in the control group if they had a systolic blood pressure less than 130 mm Hg and/or a diastolic blood pressure less than 85 mm Hg on at least 3 separate measurements. Control subjects were enrolled from Kursk hospitals during periodic medical examinations at public institutions and industrial enterprises of the Kursk region. This group was recruited from the same population and during the same period.

The following criteria were used in the selection of SNPs: the SNP must be tagging, have a minor allele frequency of at least 0.05 in the European population and be characterized by a high regulatory potential. According to the bioinformatic tool SNPinfo Web Server (https://snpinfo.niehs.nih.gov/snpinfo/snptag.html), which was used to select SNPs based on the reference haplotypic structure of the Caucasian population (CEU) of the project HapMap, the only tag SNP in the gene *SERF2* (small EDRK-rich factor 2, ID:10169) is rs4644832.

This genetic variant is localized in the intron. Several bioinformatic resources were used to assess the regulatory potential of this SNP to justify the selection of this genetic variant according to the study inclusion criteria. According to SNPinfo Web Server and the search query “SNP Function Prediction” this SNP possess the high regulatory potential (RP=0.403) and is localized within transcription factors binding site (https://snpinfo.niehs.nih.gov/snpinfo/snpfunc.html) (accessed on 8 October 2022) (Xu and Taylor, 2009). According to rSNPBase (http://rsnp.psych.ac.cn/index.do), rs4644832 is characterized by proximal and distal regulation of transcription and RNA-binding proteins-mediated regulation (accessed on 8 October 2022) (Guo and Wang, 2018). The RegulomeDB tool showed that rs4644832 *SERF2* is characterized by a regulatory coefficient of 4 (TF binding + DNase peak) (http://regulome.stanford.edu/) (accessed on 8 October 2022) (Dong and Boyle, 2019). According to the data presented in the Ensembl genome browser, this genetic variant is characterized by an average frequency of the minor G allele in European populations of 0.18 (https://www.ensembl.org/). Thus, rs4644832 *SERF2* was selected for our research, which meets the necessary criteria for inclusion in the study.

### Genetic Analysis

DNA analysis was carried out at the Research Institute for Genetic and Molecular Epidemiology of Kursk State Medical University (Kursk, Russia). Approximately 5 mL of venous blood from the cubital vein of each participant was collected into EDTA-coated tubes and maintained at − 20 ◦C until processed. Genomic DNA was extracted from thawed blood samples by the standard procedure of phenol/chloroform extraction and ethanol precipitation. Genotyping of the SNP was done using allele-specific probe-based real‐time polymerase chain reaction assays according to the protocol designed in the Laboratory of Genomic Research, Research Institute for Genetic and Molecular Epidemiology. Primers and probes were designed using the Primer3 program online (http://primer3.ut.ee/) (Koressaar and Remm, 2007), selected, and then synthesized by the Syntol company (Moscow, Russia). The two primers were used for genotyping of the polymorphism: forward 5′-TTCCGTTCACCCTAAACACC-3′ and reverse 5′-AGGGTGGTCCCGTGAAGTAG-3′ as well as two allele-specific probes 5′-FAM-CGCTCTGTGGCCACCCTCA-RTQ1–3′ and 5′-ROX-CGCTCTGTAGCCACCCTCA-BHQ2–3′. A real-time PCR was done in a 25-mL reaction mixture containing 1.5 U of Hot Start Taq DNA polymerase (Biolabmix, Novosibirsk, Russia), about 1 μg of DNA, 0.25 μM each primer, 250 μM dNTPs, 3.0 mM MgCl_2_, 1xPCR buffer [67 mM Tris-HCl, pH 8.8, 16.6 mM (NH4)_2_SO_4_, 0.01% Tween-20]. The amplification reaction consisted of an initial denaturation for 10 min at 95 °C, followed by 39 cycles of 92 °C for 30 s and 64 °C for 1 min. Fig. S1 of supplementary shows allelic discrimination plot for SNP rs4644832 *SERF2* assay designed for this study. To ensure quality control, 10% of DNA samples were genotyped in duplicates blinded to the case-control status. The concordance rate was > 99%.

### Statistical and Bioinformatics Analysis

All statistical analyses were performed in R software v3.6.3. The distribution of quantitative data was tested for normality using Shapiro–Wilk’s test (“normtest” package) and variance equality was assessed using Levene’s test (“lawstat” package). For quantitative variables, the significance of the difference between means was determined using Wilcoxon-Mann-Whitney test in case of pairwise comparison or Kruskal-Wallis rank sum test in case of multiply comparison. For categorical variables, the statistical significance of differences was evaluated by Pearson’s chi-squared test with Yates’s correction for continuity.

Compliance of the genotypes’ distribution with Hardy-Weinberg equilibrium was assessed using Fisher’s exact test. Genotype frequencies in the study groups and their associations with the disease risk were analyzed using SNPStats software (https://www.snpstats.net/start.htm) (Sole et al., 2006). For the analysis of associations of genotypes, additive models were considered. Associations were adjusted for age, gender and smoking status.

### The following bioinformatics resources were used to analyze the functional effects of rs4644832 *SERF2:*

• GTExportal (http://www.gtexportal.org/) was used to analyze the expression levels of the studied genes in brain, whole blood, and blood vessels, as well as to analyze the binding of SNPs to quantitative expression trait loci (eQTLs) (accessed on 8 October 2022) (The GTEx Consortium, 2020).
• Bioinformatics resource HaploReg (v4.1) (http://archive.broadinstitute.org/mammals/haploreg/haploreg.php) was used to assess the association of rs4644832 *SERF2* with histone modifications marking promoters and enhancers: mono-methylation at the 4th lysine residue of the histone H3 protein (H3K4me1), tri-methylation at the 4th lysine residue of the histone H3 protein (H3K4me3), the acetylation at the 9th lysine residues of the histone H3 protein (H3K9ac), acetylation of the lysine residues at N-terminal position 27 of the histone H3 protein (H3K27ac). This resource has also been used to analyze the localization of SNPs in regions of DNase hypersensitivity, regions of regulatory motifs, and sites binding with regulatory proteins (accessed on 8 October 2022) (Ward and Kellis, 2016).
• Bioinformatic tools of GeneMANIA (http://genemania.org) were utilized for the search of the main functional partners of *SERF2* (accessed on 8 October 2022) (Franz et al., 2018). Analysis of pathological pathways reflecting interactions with main functionally related proteins was carried out in Enrichr (https://amp.pharm.mssm.edu/Enrichr/) (Xie et al., 2021).
• For bioinformatics analysis of interactions between the *SERF2* gene and cigarette smoke components, the Comparative Toxicogenomics Database resource (CTD) available online, was used (http://ctdbase.org) (accessed on 10 October 2022) (Davis et al., 2021). CTD provides the ability to analyze specific interactions between genes and chemicals in vertebrates and invertebrates based on data obtained from published scientific studies worldwide. This tool was used to analyze binary interactions involving one chemical and one gene/protein.
• The Cerebrovascular Disease Knowledge Portal (CDKP) (https://cd.hugeamp.org/) was used for bioinformatic analyze of the associations of rs4644832 *SERF2* with atherosclerosis-associated diseases, intermediate phenotypes, and risk factors for IS (such as blood pressure, heart rate etc.) (accessed on 10 October 2022) (Crawford et al., 2018).

## Results

### rs4644832 is associated with IS risk in females and non-smokers

The frequency of the minor allele G in Russians (0.156) was within the range of frequencies for this allele in European populations from the 1000 Genomes Project, Phase 3 (http://www.ensembl.org (accessed on 11 October 2022): 0.154–0.222).

The genotype frequencies of rs4644832 *SERF2* in study groups are presented in Table 2. The distribution of genotypes frequencies corresponded to the Hardy-Weinberg equilibrium in the control group (P > 0.05). However, patients with IS showed an increase in observed heterozygosity (Ho=0.3136) compared to expected (He=0.2877); P < 0.01.

**Table 2:** Results of the analysis of associations between rs4644832 (A/G) *SERF2* and ischemic stroke risk.

Genotypes	Controls (N = 1068)	IS patients (N = 861)	OR (95% CI)	P
A/A	765 (71.6%)	576 (66.9%)	1.17 (0.98–1.40)^1^	0.089^2^
A/G	272 (25.5%)	270 (31.4%)		
G/G	31 (2.9%)	15 (1.7%)		
Maf (G)	0.156	0.174		
Males (controls (N = 468)/IS patients (N = 480))				
A/A	345 (73.7%)	340 (70.8%)	1.06 (0.82–1.36)	0.68
A/G	110 (23.5%)	134 (27.9%)		
G/G	13 (2.8%)	6 (1.2%)		
Maf (G)	0.145	0.152		
Females (controls (N = 600)/IS patients (N = 381))				
A/A	420 (70%)	236 (61.9%)	**1.29 (1.02–1.64)**	**0.035**
A/G	162 (27%)	136 (35.7%)		
G/G	18 (3%)	9 (2.4%)		
Maf (G)	0.165	0.202		
Non-smokers (controls (N = 750)/IS patients (N = 432))				
A/A	532 (70.9%)	275 (63.7%)	**1.26 (1.01–1.56)**	**0.041**
A/G	194 (25.9%)	146 (33.8%)		
G/G	24 (3.2%)	11 (2.5%)		
Maf (G)	0.161	0.194		
Smokers (controls (N = 318/IS patients (N = 429))				
A/A	233 (73.3%)	301 (70.2%)	1.08 (0.80–1.45)	0.61
A/G	78 (24.5%)	124 (28.9%)		
G/G	7 (2.2%)	4 (0.9%)		
Maf (G)	0.145	0.154		

^1^ – odds ratio and 95% confidence interval adjusted by sex, age; ^2^ – P-value adjusted by sex, age. All calculations were performed relative to the minor allele G. Statistically significant differences are marked in bold.

The analysis of the total sample did not reveal significant associations between rs4644832 polymorphism *SERF2* and IS risk. However, sex-stratified analysis demonstrated that G allele is the risk factor of IS in females (OR=1.29, 95%CI 1.02–1.64, P = **0.035**). Also, this genetic variant was found to be associated with an increased risk of IS in non-smoking individuals (OR=1.26, 95%CI 1.01–1.56, P = **0.041**) (Table 2).

Since the revealed association between the genetic polymorphism and smoking-status may be biased by the disequilibrium of proportion of smokers between males and females we further performed a separated analysis accounting both factors. The analysis has confirmed that smoking-status has its own impact because we had found that the association of G allele with the higher risk of IS in nonsmokers still took place even after exclusion of males (Table 3).

**Table 3:** Results of the analysis of associations between rs4644832 (A/G) *SERF2* and ischemic stroke risk depend on sex and smoking status.

Genotypes	Controls (N = 1068)	IS patients (N = 861)	OR (95% CI)	P
Males/nonsmokers (controls (N = 220)/IS patients (N = 184))				
A/A	157 (71.4%)	123 (66.8%)	1.16 (0.79–1.68)^1^	0.45^2^
A/G	57 (25.9%)	57 (31%)		
G/G	6 (2.7%)	4 (2.2%)		
Maf (G)	0.157	0.177		
Males/smokers (controls (N = 248)/IS patients (N = 296))				
A/A	188 (75.8%)	217 (73.3%)	1.02 (0.71–1.44)	0.93
A/G	53 (21.4%)	77 (26%)		
G/G	7 (2.8%)	2 (0.7%)		
Maf (G)	0.135	0.137		
Females/nonsmokers (controls (N = 530)/IS patients (N = 248))				
A/A	375 (70.8%)	152 (61.3%)	1.34 (1.02–1.76)	**0.035**
A/G	137 (25.9%)	89 (35.9%)		
G/G	18 (3.4%)	7 (2.8%)		
Maf (G)	0.163	0.208		
Females/smokers (controls (N = 70)/IS patients (N = 133))				
A/A	45 (64.3%)	84 (63.2%)	1.11 (0.62–1.98)	0.72
A/G	25 (35.7%)	47 (35.3%)		
G/G	0 (0%)	2 (1.5%)		
Maf (G)	0.179	0.192		

^1^ – odds ratio and 95% confidence interval; ^2^ – P-value. All calculations were performed relative to the minor allele G. Statistically significant differences are marked in bold.

Given the fact that patients with IS were characterized by differences in clinical and laboratory characteristics depending on the presence of a comorbid pathology (IHD/T2DM), there may be data bias in the results of associations. Therefore, in order to understand the marker specificity for IS, we considered comparison of the 3 patients’ subgroups depending on the presence/absence of other diseases: stroke patients without IHD and T2DM, combination of stroke and CAD, stroke patients with T2DM (Tables S2-S4). Of note, we did not segregate a subgroup of stroke patients aggravated by both CHD and T2DM in the analysis due to the small number of observations in this cohort. The association of rs4644832 with the IS in non-smoking patients persisted after the exclusion of stroke patients with comorbid IHD and T2DM. However, the association of this genetic variant with IS in women was lost, possibly due to a reduction in study power due to a decrease in the number of patients by 207 people. We did not find associations of rs4644832 *SERF2* in the subgroups of patients with IHD and T2DM, which is an additional justification for the specific association of rs4644832 *SERF2* with the IS development. On the other hand, the number of stroke patients with comorbid IHD and T2DM was too small to draw any conclusions about associations with genetic markers.

### Molecular correlates of rs4644832 *SERF2*

**Fig. 1:**
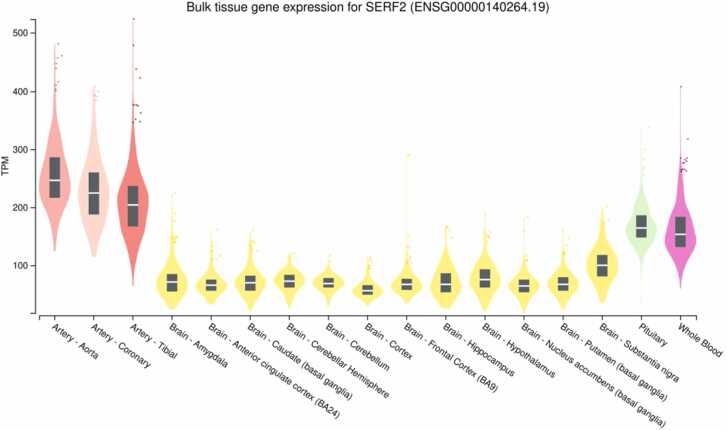
Level of the expression of the *SERF2* gene in brain, whole blood, and blood vessels (https://gtexportal.org).

The *SERF2* gene is expressed in brain tissues, blood vessels, and whole blood. In brain tissues, gene expression levels of *SERF2* measured as median (Me) TPM (Transcripts Per Million) vary from 56.54 to 164.7; in blood vessels – from 203.8 to 246.5; in whole blood MeTPM= 153.5 (Fig. 1).

The protective A allele of rs4644832 upregulates *SERF2* expression in blood vessels and in whole blood (Table S5). This genetic variant alters cis-eQTL-mediated regulation of the expression of 10 other genes (*AC011330.5, ADAL, CATSPER2, CATSPER2P1, HYPK, MAP1A, PDIA3, STRC, STRCP1, ZSCAN29*) in the brain, blood vessels, and whole blood. Besides the others, the list of *SERF*-regulated genes includes *HYPK* (Huntingtin Interacting Protein K), involved in negative regulation of apoptosis and protein stabilization; *MAP1A* (Microtubule Associated Protein 1A), *PDIA3* (molecular chaperone that prevents the formation of protein aggregates) (Table S5).

**Fig. 2:**
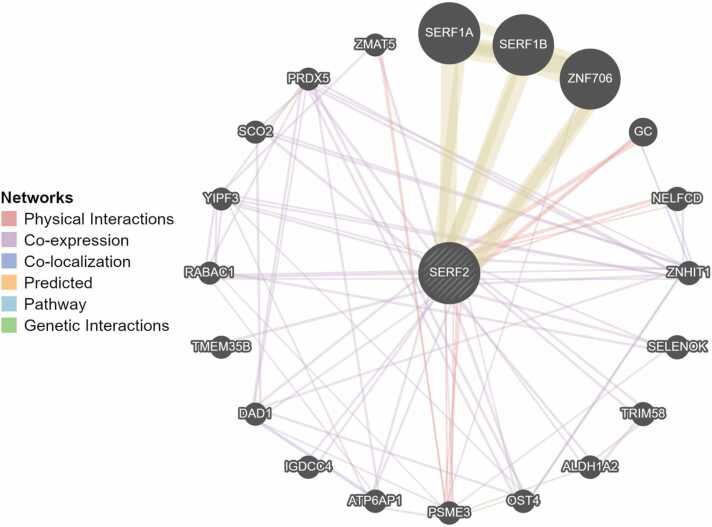
Interactome web reflecting protein-protein interactions of the main functional partners of SERF2 (extracted from GeneMANIA).

The SERF2 protein is associated with 20 functional partners, and 92 total links the most significant of which are SERF1A, ZNF706, SERF1B (Fig. 2). Together with SERF2, these proteins are involved in such general biological processes as protein destabilization (GO:0031648), Padj= 0.0002; regulation of stem cell population maintenance (GO:2000036), Padj= 0.02; amyloid fibril formation (GO:1990000), Padj= 0.04 (https://maayanlab.cloud/Enrichr).

Subsequent analysis revealed a significant effect of rs4644832 *SERF2* on histone modifications. This genetic variant is located in the region of DNA binding to histone H3, characterized by mono-methylation at the 4th lysine residue of the histone H3 protein (H3K4me1) and marking enhancers in Brain Hippocampus Middle, Brain Cingulate Gyrus, Cells from peripheral blood, as well as tri-methylation at the 4th lysine residue of the histone H3 protein (H3K4me3) and marking promoters in all brain tissues represented by HaploReg (v4.1), and Cells from peripheral blood. The effect of this histone mark is enhanced by acetylation of the lysine residues at N-terminal position 27 of the histone H3 protein (H3K27ac), marking enhancers in peripheral blood cells and all brain tissues, as well as the acetylation at the 9th lysine residues of the histone H3 protein (H3K9ac), marking promoters in blood cells and all brain tissues, except Brain Hippocampus Middle (Table 4).

**Table 4:** Tissue-specific effects of rs4644832 *SERF2* on histone modifications (https://pubs.broadinstitute.org/mammals/haploreg/haploreg.php).

Tissues	H3K4me1	H3K4me3	H3K27ac	H3K9ac
Brain Hippocampus Middle	Enh	Pro	Enh	–
Brain Substantia Nigra	–	Pro	Enh	Pro
Brain Anterior Caudate	–	Pro	Enh	Pro
Brain Cingulate Gyrus	Enh	Pro	Enh	Pro
Brain Inferior Temporal Lobe	–	Pro	Enh	Pro
Brain Angular Gyrus	–	Pro	Enh	Pro
Brain_Dorsolateral_Prefrontal_Cortex	–	Pro	Enh	Pro
Cells from peripheral blood (any)	Enh	Pro	Enh	Pro

H3K4me1 – mono-methylation at the 4th lysine residue of the histone H3 protein; H3K4me3 – tri-methylation at the 4th lysine residue of the histone H3 protein; H3K9ac – the acetylation at the 9th lysine residues of the histone H3 protein; H3K27ac – acetylation of the lysine residues at N-terminal position 27 of the histone H3 protein; Enh – histone modification in the enhancer region; Pro – histone modification at the promoter region.

According to the bioinformatic resource Cardiovascular Disease Knowledge Portal (CVDKP, https://cvd.hugeamp.org), which combines and analyzes the results of genetic associations of the largest consortiums for the study of CVD, the protective allele A is associated with a number of stroke-associated phenotypes (Table 5).

**Table 5:** Summarized analysis of associations between rs4644832 *SERF2* and stroke-associated phenotypes (data obtained from Cardiovascular Disease Knowledge Portal, https://cvd.hugeamp.org/).

№	SNP	Phenotype	P-Value	Beta (OR)	Sample Size
	rs4644832 *SERF2* (G/A)	Diastolic blood pressure	7.96 × 10^−10^	_Beta_↓ − 0.0152	1 183 530
		Triglycerides	1.09 × 10^−9^	_Beta_↑ 0.0105	1 926 180
		Heart rate	2.04 × 10^−4^	_Beta_↓ − 0.0915	484 178
		Systolic blood pressure	0.001	_Beta_↓ − 0.0074	1 169 900
		Non-HDL cholesterol	0.02	_Beta_↑ 0.0053	1 043 500
		Hypertension	0.02	_OR_↓ 0.9894	505 242
		Peripheral artery disease in ever smokers	0.03	_OR_↓ 0.9500	28 235
		HDL cholesterol	0.049	_Beta_↓ − 0.0031	1 618 710

Effect: increase (↑) or decrease (↓) gene expression. All calculations were performed relative to the major allele A

## Discussion

Preserving proteome quality is one of the primary tasks for the cell. Recently described class of chaperones, Hero, obviously provides important homeostatic functions and should be taken into account for its role in pathogenesis of various pathologies. Hero proteins were discovered in 2020 as heat-resistant molecules responsible for chaperone-like activity of the supernatants collected from the boiled S2 or HEK293T cell cultures lysates (Tsuboyama et al., 2020). Thorough search for the nature of protein-preserving components of these boiled supernatants allowed identifying six proteins which were shown to protect proteins under stress conditions and to prevent pathogenic misfolded TDP-43 aggregations in cells. The authors also demonstrated that the hallmarks of the novel class of chaperones were low molecular weight, high polarity and charge, as well as disorganized structure. The most obvious way to explain their chaperone activity is that because of chemical properties they are able to physically screen client proteins.

Since Hero-proteins had been demonstrated as substantial regulators of proteostasis we aimed to investigate whether their member SERF2 is involved in pathogenesis of IS. Previously, SERF2 was reported to enhance polyglutamine, β-amyloid and α-Synuclein aggregation and to contribute to amyloid proteotoxicity ([49], [17], [33]). Moreover, brain-specific *Serf2* knockout mice, though viable, appear to be more prone to deposition of amyloids, and show modified fibril morphology (Stroo et al., 2021). Whole-body knockouts are perinatally lethal due to an apparently unrelated developmental issue (Cleverley et al., 2021). A number of works reported that SERF2 interacts with a wide spectrum of RNA molecules, however the physiological significance or specificity of this interaction is unclear at this time (Sahoo and Bardwell, 2022).

Here, providing genetic evidence that rs4644832 is associated with IS risk, we report *SERF2* is involved in pathogenesis of stroke. Probably, the role of *SERF2* in IS may be related to some of its protective functions because disease-causing allele G was shown to decrease *SERF2* expression. In respect of main causes of IS these protective effects are most likely related to the role of *SERF2* in the cardiovascular system. This assumption is further enhanced by previous findings confirming correlation between rs4644832 polymorphism and different cardiovascular phenotypes. For instance, CDKP demonstrates that protective A allele is associated with a decrease in Diastolic blood pressure (Beta=−0.0152, P = 7.96 ×10^−10^), Systolic blood pressure (Beta=−0.0074, P = 0.001) as well as with a risk of Arterial hypertension (OR=0.9894, P = 0.02) (Table 5).

Despite it seems difficult to separate the impact of rs4644832 on the risk of IS or of other related cardiovascular diseases, we report that genetic association remains significant even after excluding IHD and DM type 2 patients. Nevertheless, all the patients had confirmed arterial hypertension in anamnesis. Thus, bioinformatic analysis and our results indicate that this genetic variant may play a significant role in the rise of hypertension – the most important factor contributing to IS risk. Differentiation of rs4644832 contribution to isolated arterial hypertension is one of the main limitations in the present study and should be considered the task for further research. We also have to note that another limitation of our research is missing values of some clinical parameters in a certain number of patients (Table 1, Table S1) due to the retrospective type of the study.

Additionally, CDKP shows that rs4644832 A allele is protective for Elevated heart rate (Beta=−0.0915, P = 2.04 ×10^−4^) and Peripheral artery disease (OR=0.95, P = 0.03). Elevated heart rate increases the risk of atrial fibrillation acting as another significant risk factor for cardioembolic type of IS. Notably, CDKP data also indicates A allele to have a protective role in peripheral artery disease – pathogenetically related cardiovascular pathology. Noteworthy, all these associations are concordant to pretty high levels of *SERF2* expression in cardiovascular system, however precise molecular avenues of *SERF2* involvement in the listed phenotypes are yet to be understood in details. Obviously, of the main mechanisms, chaperone-like or RNA-binding activity should be considered first.

Revealed sensitivity to mutation for only women and non-smokers may be theoretically related to abolishment of protective effects of the A allele for males and smokers who are more prone to cardiovascular diseases. However, previous data show that in contract to estrogens (Gertz et al., 2012) tobacco components ([44], [26], [22]) and androgens (Bereketoglu et al., 2021) serve positive regulators of *SERF2* expression making a hint that these sex/smoking-gene relationships may still be explained by cellular effects of SERF2 only. Taking together our data suggest that *SERF2* has a protective role because the factors increasing its expression (male sex, smoking, A allele) decrease the risk of IS. Moreover, our previous results showing correlation between rs2900262 polymorphism of another Hero gene – *C9ORF16* with IS only in smoking individuals also match this tendency because components of cigarette smoke, on the contrary, reduce the expression of *C9ORF16* (Kobzeva et al., 2022).

Interestingly, in an attempt to bridge *SERF2* function and IS we have found that of the genes influenced by eQTL effects of rs4644832 none had previously been shown to correlate with cardiovascular phenotypes. However, it turned out that some of them possess great impact for neurodegeneration and protein quality control. For instance, *MAP1A,* regulative by rs4644832, encodes a neurospecific protein which is known as a causative for neurodegenerative diseases (https://www.genecards.org/cgi-bin/carddisp.pl?gene=MAP1A&keywords=MAP1A, Liu et al., 2015). SNP rs4644832 is also correlated to expression of *PDIA3*, the gene encoding a protein of the endoplasmic reticulum that interacts with lectin chaperones calreticulin and calnexin to modulate folding of newly synthesized glycoproteins (Tatusova et al., 2016). *PDIA3* сatalyzes the formation, isomerization, and reduction or oxidation of disulfide bonds ([3], [19]), playing an important role in post-translational modification and folding of client proteins. Another gene, *HYPK*, is involved in negative regulation of apoptosis regulation and protein stabilization (Raychaudhuri et al., 2008). Moreover, *HYPK* has the chaperone-like activity and is related to regulation of heat shock response (Das and Bhattacharyya, 2016) and protein homeostasis (Ghosh and Ranjan, 2022). Altogether, these findings open up future perspectives to study possible links between *SERF2* and outcomes of IS because proteostasis takes an especially important place during response to ischemia-reperfusion.

## Conclusion

The present study reveals the novel genetic association between rs4644832 *SERF2* and the risk of IS in females and non-smokers. These data provide the new insights of involvement of recently discovered class of chaperones Hero in both neurological and cardiovascular pathology. Further studies may help to uncover the details of its particular role in the pathogenesis of stroke.

## Compliance with ethical standards

The study was conducted according to the guidelines of the Declaration of Helsinki, and was approved by the Ethical Review Committee of Kursk State Medical University, Russia (Protocol No. 12 from 7.12.2015). All the participants gave written informed consent before the enrollment in this study.

## Funding Information

This research was funded by the 10.13039/501100006769Russian Science Foundation [№ 22–15–00288, https://rscf.ru/en/project/22–15–00288/].

## Patient consent for publication

Not applicable.

## Consent for publication

All authors have read and accepted responsibility for the content of the manuscript.

## CRediT authorship contribution statement

A.E.B. wrote the initial draft. K.A.K., M.O.S., T.A.S. collected the data, conducted experiments. M.B.F. and A.V.P. participated in the conceptualization, data processing, formal analysis, and project writing. V.O.S. and O.Y.B. conceived and developed the study. M.I.C. and A.E.B. participated in the formal analysis, data analysis, research, and methodology. V.O.S., O.Y.B., M.B.F. and A.V.D. were involved in data analysis and interpretation, data curation, funding, oversight, and original writing. O.Y.B., M.B.F. analyzes and interprets data, project management, supervision and review and editing. K.A.K., T.A.S. and O.Y.B. confirm the authenticity of all raw data. All authors read and approved the final manuscript.

## Declaration of Competing Interest

The authors declare no conflict of interests.
