# Supplementary material for: Polymorphism of SERF2, the gene encoding a heat-resistant obscure (Hero) protein with chaperone activity, is a novel link in ischemic stroke
Source: IBRO Neurosci Rep. 2023 May 10;14:453–61. doi: 10.1016/j.ibneur.2023.05.004 (PMC10209486; doi:10.1016/j.ibneur.2023.05.004)
Supplement: Supplementary file 1 — Supplementary material [file mmc1.docx]

**Supplementary materials:**


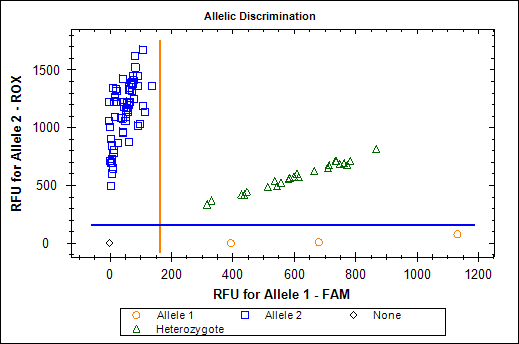


**Fig. S1.** Allelic discrimination plot for SNP rs4644832 *SERF2* assay designed for this study. The plot shows clear separation between the signals derived from allele 1 (rs4644832 G, FAM fluorescent dye) or allele 2 rs4644832 A, ROX fluorescent dye). Genotypes AA, AG, and GG are shown as squares, triangles, and circles, respectively.

**Table S1**

Baseline and clinical characteristics of the subgroups of patients depending on the presence/absence of comorbid diseases

| Baseline and clinical characteristics | | IS  (n=669) | IS + DM  (n=80) | IS + IHD  (n=97) | IS + DM + IHD  (n=15) | P |
| --- | --- | --- | --- | --- | --- | --- |
| Age, Ме [Q1; Q3] | | 61 [54; 68] | 64.5 [55; 69.25] | 65 [60; 72] | 70 [62.5; 75] | **<0.001** |
| Gender | Males, N(%) | 371 (55.5%) | 39 (48.8%) | 63 (64.9%) | 7 (46.7%) | 0.14 |
|  | Females, N(%) | 298 (44.5%) | 41 (51.2%) | 34 (35.1%) | 8 (53.3%) |  |
| Smoking | Yes, N (%) | 351 (52.5%) | 30 (37.5%) | 42 (43.3%) | 6 (40%) | **<0.05** |
|  | No, N (%) | 318 (47.5%) | 50 (62.5%) | 55 (56.7%) | 9 (60%) |  |
| Body mass index, Ме [Q1; Q3] | | 23 [22; 24]  (n=425) | 24 [22; 28]  (n=67) | 25 [23; 29]  (n=57) | 25 [23.75; 29.25]  (n=12) | **<0.001** |
| Family history of cerebrovascular diseases | Yes, N (%) | 236 (35.7) | 26 (35.1) | 34 (37.4) | 5 (41.7) | 0.96 |
|  | No, N (%) | 425 (64.3) | 48 (64.9) | 57 (62.6) | 7 (58.3) |  |
| Age at onset of stroke, Ме [Q1; Q3] | | 60 [53; 67.5]  (n=655) | 61 [53; 70]  (n=80) | 64.5 [58.25; 72]  (n=90) | 70 [61; 76.5]  (n=15) | **<0.001** |
| Number of strokes including event in question | 1, N (%) | 593 (90.5%) | 69 (86.2%) | 76 (85.4%) | 13 (86.7%) | 0.36 |
|  | 2, N (%) | 54 (8.2%) | 10 (12.5%) | 11 (12.4%) | 1 (6.7%) |  |
|  | 3, N (%) | 8 (1.2%) | 1 (1.2%) | 2 (2.2%) | 1 (6.7%) |  |
| Stroke localization | Right/left middle cerebral artery basin,  N (%) | 546 (83.4%) | 69 (86.2%) | 74 (83.1%) | 12 (80%) | 0.90 |
|  | Vertebrobasilar basin,  N (%) | 109 (16.6%) | 11 (13.8%) | 14 (15.7%) | 3 (20%) |  |
| Area of lesion in stroke, mm^2^, Ме [Q1; Q3] | | 102 [25; 460]  (n=649) | 80.5 [30.5; 268]  (n=78) | 150 [50.5; 421.5]  (n=87) | 500 [61; 1010.5]  (n=15) | 0.25 |
| Total cholesterol, mmol/L, Ме [Q1; Q3] | | 5.1 [4.4; 5.8]  (n=425) | 5.6 [4.95; 6.1]  (n=67) | 5.4 [4.35; 5.9]  (n=60) | 6.05 [5.65; 6.65]  (n=12) | **<0.01** |
| Triglycerides, mmol/L, Ме [Q1; Q3] | | 1.3 [1; 1.7]  (n=422) | 1.5 [1.2; 2.2]  (n=67) | 1.3 [1; 1.7]  (n=58) | 1.75 [1.3; 2.2]  (n=12) | **<0.01** |
| Glucose level, mmol/L, Ме [Q1; Q3] | | 4.6 [4.3; 5.4]  (n=654) | 8.1 [5.35; 8.1]  (n=80) | 4.8 [4.2; 5.5]  (n=92) | 6.6 [4.7; 8.1]  (n=15) | **<0.001** |
| Prothrombin time, seconds, Ме [Q1; Q3] | | 10.66 [10.14; 11.7]  (n=648) | 11.31 [10.14; 12.35]  (n=77) | 11.31 [10.14; 13.26]  (n=86) | 12.35 [9.68; 13.65]  (n=15) | **<0.05** |
| International normalized ratio, Ме [Q1; Q3] | | 1 [0.94; 1.08]  (n=421) | 1 [0.91; 1.03]  (n=64) | 1 [0.99; 1.1]  (n=52) | 1 [0.9; 1]  (n=12) | 0.13 |
| Activated partial thromboplastin time, seconds, Ме [Q1; Q3] | | 32.4 [29; 37]  (n=421) | 32.9 [30.02; 36]  (n=66) | 35 [31; 37.1]  (n=53) | 32.45 [31; 35.5]  (n=12) | 0.69 |
| Alanin aminotransferase, IU/L | | 21.9 [18; 31.2]  (n=543) | 22.25 [20; 53.55]  (n=54) | 22 [16.73; 41.4]  (n=42) | 24.4 [23.3; 45.65]  (n=7) | 0.26 |
| Aspartate aminotransferase, IU/L | | 25.6 [19.8; 37.7]  (n=543) | 32.1 [23; 36.58]  (n=54) | 28.35 [21.4; 34.3]  (n=42) | 25.5 [24.5; 38.3]  (n=7) | 0.71 |
| Statistically significant differences between groups are indicated in bold; IS – ischemic stroke, T2MD – type 2 diabetes mellitus, IHD – ischemic heart disease. | | | | | | |

**Table S2**

Results of the analysis of associations between rs4644832 (A/G) *SERF2* and ischemic stroke risk

(in patients without CAD and type 2 diabetes mellitus)

| Genotypes | Controls (N=1068) | IS patients (N=669) | OR (95% CI) | P |
| --- | --- | --- | --- | --- |
| A/A | 765 (71.6%) | 444 (66.4%) | 1.19 (0.98-1.45) | 0.075 |
| A/G | 272 (25.5%) | 214 (32%) |  |  |
| G/G | 31 (2.9%) | 11 (1.6%) |  |  |
| Maf (G) | 0.156 | 0.176 |  |  |
| Males (controls (N=468)/IS patients (N=371)) | | | | |
| A/A | 345 (73.7%) | 257 (69.3%) | 1.13 (0.86-1.47) | 0.39 |
| A/G | 110 (23.5%) | 109 (29.4%) |  |  |
| G/G | 13 (2.8%) | 5 (1.4%) |  |  |
| Maf (G) | 0.145 | 0.160 |  |  |
| Females (controls (N=600)/IS patients (N=298)) | | | | |
| A/A | 420 (70%) | 187 (62.8%) | 1.24 (0.96-1.61) | 0.098 |
| A/G | 162 (27%) | 105 (35.2%) |  |  |
| G/G | 18 (3%) | 6 (2%) |  |  |
| Maf (G) | 0.165 | 0.196 |  |  |
| Non-smokers (controls (N=750)/IS patients (N=318)) | | | | |
| A/A | 532 (70.9%) | 198 (62.3%) | **1.32 (1.04-1.68)** | **0.022** |
| A/G | 194 (25.9%) | 111 (34.9%) |  |  |
| G/G | 24 (3.2%) | 9 (2.8%) |  |  |
| Maf (G) | 0.161 | 0.203 |  |  |
| Smokers (controls (N=318/IS patients (N=351)) | | | | |
| A/A | 233 (73.3%) | 246 (70.1%) | 1.07 (0.78-1.46) | 0.68 |
| A/G | 78 (24.5%) | 103 (29.3%) |  |  |
| G/G | 7 (2.2%) | 2 (0.6%) |  |  |
| Maf (G) | 0.145 | 0.152 |  |  |
| ^1^ – odds ratio and 95% confidence interval adjusted by sex, age; ^2^ – P-value adjusted by sex, age. All calculations were performed relative to the minor allele G. Statistically significant differences are marked in bold. | | | | |

**Table S3**

Results of the analysis of associations between rs4644832 (A/G) *SERF2* and ischemic stroke risk

(in patients with comorbid CAD)

| Genotypes | Controls (N=1068) | IS patients (N=112) | OR (95% CI) | P |
| --- | --- | --- | --- | --- |
| A/A | 765 (71.6%) | 80 (71.4%) | 1.02 (0.69-1.50) | 0.93 |
| A/G | 272 (25.5%) | 28 (25%) |  |  |
| G/G | 31 (2.9%) | 4 (3.6%) |  |  |
| Maf (G) | 0.156 | 0.161 |  |  |
| Males (controls (N=468)/IS patients (N=70)) | | | | |
| A/A | 345 (73.7%) | 55 (78.6%) | 0.77 (0.45-1.32) | 0.33 |
| A/G | 110 (23.5%) | 14 (20%) |  |  |
| G/G | 13 (2.8%) | 1 (1.4%) |  |  |
| Maf (G) | 0.145 | 0.114 |  |  |
| Females (controls (N=600)/IS patients (N=42)) | | | | |
| A/A | 420 (70%) | 25 (59.5%) | 1.56 (0.93-2.62) | 0.1 |
| A/G | 162 (27%) | 14 (33.3%) |  |  |
| G/G | 18 (3%) | 3 (7.1%) |  |  |
| Maf (G) | 0.165 | 0.238 |  |  |
| Non-smokers (controls (N=750)/IS patients (N=64)) | | | | |
| A/A | 532 (70.9%) | 46 (71.9%) | 0.96 (0.59-1.57) | 0.88 |
| A/G | 194 (25.9%) | 16 (25%) |  |  |
| G/G | 24 (3.2%) | 2 (3.1%) |  |  |
| Maf (G) | 0.161 | 0.156 |  |  |
| Smokers (controls (N=318/IS patients (N=48)) | | | | |
| A/A | 233 (73.3%) | 34 (70.8%) | 1.18 (0.66-2.09) | 0.58 |
| A/G | 78 (24.5%) | 12 (25%) |  |  |
| G/G | 7 (2.2%) | 2 (4.2%) |  |  |
| Maf (G) | 0.145 | 0.167 |  |  |
| ^1^ – odds ratio and 95% confidence interval adjusted by sex, age; ^2^ – P-value adjusted by sex, age. All calculations were performed relative to the minor allele G. Statistically significant differences are marked in bold. | | | | |

**Table S4**

Results of the analysis of associations between rs4644832 (A/G) *SERF2* and ischemic stroke risk

(in patients with comorbid type 2 diabetes mellitus)

| Genotypes | Controls (N=1068) | IS patients (N=95) | OR (95% CI) | P |
| --- | --- | --- | --- | --- |
| A/A | 765 (71.6%) | 65 (68.4%) | 0.95 (0.63-1.44) | 0.82 |
| A/G | 272 (25.5%) | 30 (31.6%) |  |  |
| G/G | 31 (2.9%) | 0 (0%) |  |  |
| Maf (G) | 0.156 | 0.158 |  |  |
| Males (controls (N=468)/IS patients (N=46)) | | | | |
| A/A | 345 (73.7%) | 35 (76.1%) | 0.81 (0.42-1.53) | 0.5 |
| A/G | 110 (23.5%) | 11 (23.9%) |  |  |
| G/G | 13 (2.8%) | 0 (0%) |  |  |
| Maf (G) | 0.145 | 0.120 |  |  |
| Females (controls (N=600)/IS patients (N=49)) | | | | |
| A/A | 420 (70%) | 30 (61.2%) | 1.22 (0.72-2.06) | 0.47 |
| A/G | 162 (27%) | 19 (38.8%) |  |  |
| G/G | 18 (3%) | 0 (0%) |  |  |
| Maf (G) | 0.165 | 0.194 |  |  |
| Non-smokers (controls (N=750)/IS patients (N=59)) | | | | |
| A/A | 532 (70.9%) | 38 (64.4%) | 1.12 (0.69-1.82) | 0.65 |
| A/G | 194 (25.9%) | 21 (35.6%) |  |  |
| G/G | 24 (3.2%) | 0 (0%) |  |  |
| Maf (G) | 0.161 | 0.178 |  |  |
| Smokers (controls (N=318/IS patients (N=36)) | | | | |
| A/A | 233 (73.3%) | 27 (75%) | 0.84 (0.40-1.76) | 0.65 |
| A/G | 78 (24.5%) | 9 (25%) |  |  |
| G/G | 7 (2.2%) | 0 (0%) |  |  |
| Maf (G) | 0.145 | 0.125 |  |  |
| ^1^ – odds ratio and 95% confidence interval adjusted by sex, age; ^2^ – P-value adjusted by sex, age. All calculations were performed relative to the minor allele G. Statistically significant differences are marked in bold. | | | | |

**Table S5**

Effect of A allele of rs4644832 *SERF2* on gene expression (cis-eQTL) in various tissues (according to the GTEx Portal browser, <https://gtexportal.org>).

| Gene Expressed | P-Value | Effect (NES) | Tissue |
| --- | --- | --- | --- |
| *AC011330.5* | **5.6×10^-11^** | ↑(0.78) | Brain - Cerebellum |
| *AC011330.5* | **4.7×10^-9^** | ↑(0.72) | Brain - Cerebellar Hemisphere |
| *AC011330.5* | **3.0×10^-8^** | ↑(0.67) | Brain - Caudate (basal ganglia) |
| *AC011330.5* | **3.3×10^-8^** | ↑(0.72) | Brain - Anterior cingulate cortex (BA24) |
| *AC011330.5* | **3.9×10^-7^** | ↑(0.58) | Pituitary |
| *AC011330.5* | **4.4×10^-7^** | ↑(0.45) | Artery - Aorta |
| *AC011330.5* | **8.3×10^-7^** | ↑(0.65) | Brain - Hippocampus |
| *AC011330.5* | **3.2×10^-6^** | ↑(0.59) | Brain - Frontal Cortex (BA9) |
| *AC011330.5* | **3.3×10^-6^** | ↑(0.62) | Brain - Putamen (basal ganglia) |
| *AC011330.5* | **1.1×10^-5^** | ↑(0.29) | Artery - Tibial |
| *AC011330.5* | **1.1×10^-5^** | ↑(0.52) | Brain - Cortex |
| *AC011330.5* | **3.7×10^-5^** | ↑(0.50) | Artery - Coronary |
| *AC011330.5* | **2.3×10^-4^** | ↑(0.41) | Brain-Nucleus accumbens (basal ganglia) |
| *ADAL* | **7.6×10^-6^** | ↓(-0.24) | Artery - Tibial |
| *CATSPER2* | **7.7×10^-7^** | ↑(0.45) | Brain - Cerebellum |
| *CATSPER2* | **4.0×10^-6^** | ↓(-0.21) | Artery - Tibial |
| *CATSPER2P1* | **1.3×10^-5^** | ↓(-0.27) | Artery - Tibial |
| *HYPK* | **2.5×10^-4^** | ↑(0.24) | Brain - Cerebellar Hemisphere |
| *MAP1A* | **7.8×10^-5^** | ↓(-0.18) | Artery - Aorta |
| *PDIA3* | **2.8×10^-5^** | ↑(0.096) | Whole Blood |
| *SERF2* | **1.1×10^-8^** | ↑(0.10) | Whole Blood |
| *SERF2* | **3.3×10^-7^** | ↑(0.17) | Artery - Aorta |
| *SERF2* | **7.9×10^-6^** | ↑(0.09) | Artery - Tibial |
| *STRC* | **5.8×10^-14^** | ↓(-0.64) | Brain - Frontal Cortex (BA9) |
| *STRC* | **1.1×10^-12^** | ↓(-0.62) | Brain - Cortex |
| *STRC* | **1.5×10^-12^** | ↓(-0.51) | Brain - Caudate (basal ganglia) |
| *STRC* | **3.9×10^-11^** | ↓(-0.52) | Brain-Nucleus accumbens (basal ganglia) |
| *STRC* | **1.4×10^-8^** | ↓(-0.38) | Brain - Putamen (basal ganglia) |
| *STRC* | **3.5×10^-8^** | ↓(-0.51) | Brain - Hypothalamus |
| *STRC* | **3.2×10^-6^** | ↓(-0.43) | Brain - Anterior cingulate cortex (BA24) |
| *STRC* | **7.5×10^-6^** | ↓(-0.30) | Artery - Aorta |
| *STRC* | **1.7×10^-5^** | ↓(-0.25) | Artery - Tibial |
| *STRCP1* | **7.2×10^-7^** | ↓(-0.28) | Artery - Tibial |
| *STRCP1* | **1.2×10^-6^** | ↓(-0.33) | Artery - Aorta |
| *ZSCAN29* | **5.0×10^-5^** | ↑(0.11) | Whole Blood |
| Effect: increase (↑) or decrease (↓) gene expression; NES – normalized effect size. | | | |
